# Electropermeabilization of Inner and Outer Cell Membranes with Microsecond Pulsed Electric Fields: Quantitative Study with Calcium Ions

**DOI:** 10.1038/s41598-017-12960-w

**Published:** 2017-10-12

**Authors:** Hanna Hanna, Agnese Denzi, Micaela Liberti, Franck M. André, Lluis M. Mir

**Affiliations:** 1 Vectorology and Anticancer Therapies, UMR 8203, CNRS, Univ. Paris-Sud, Gustave Roussy, Université Paris-Saclay, 94 805 Villejuif, France; 2 Department of Information Engineering, Electronics and Telecommunication (DIET), University of Rome “La Sapienza”, Rome, 00184 Italy

## Abstract

Microsecond pulsed electric fields (μsPEF) permeabilize the plasma membrane (PM) and are widely used in research, medicine and biotechnology. For internal membranes permeabilization, nanosecond pulsed electric fields (nsPEF) are applied but this technology is complex to use. Here we report that the endoplasmic reticulum (ER) membrane can also be electropermeabilized by one 100 µs pulse without affecting the cell viability. Indeed, using Ca^2+^ as a permeabilization marker, we observed cytosolic Ca^2+^ peaks in two different cell types after one 100 µs pulse in a medium without Ca^2+^. Thapsigargin abolished these Ca^2+^ peaks demonstrating that the calcium is released from the ER. Moreover, IP3R and RyR inhibitors did not modify these peaks showing that they are due to the electropermeabilization of the ER membrane and not to ER Ca^2+^ channels activation. Finally, the comparison of the two cell types suggests that the PM and the ER permeabilization thresholds are affected by the sizes of the cell and the ER. In conclusion, this study demonstrates that µsPEF, which are easier to control than nsPEF, can permeabilize internal membranes. Besides, μsPEF interaction with either the PM or ER, can be an efficient tool to modulate the cytosolic calcium concentration and study Ca^2+^ roles in cell physiology.

## Introduction

Cell electroporation involves the application of electric pulses in order to increase the plasma membrane (PM) permeability. This concept emerged in the mid-60s with the work of Sale and Hamilton (1968)^1^. Molecules that cannot cross the PM in normal conditions can reach the cell cytosol due to the delivery of one or several electric pulses^2^. Furthermore, in 1982, Neumann *et al*. showed that gene transfer into a cell was possible by the application of electric pulses^3^.

In the quiescent state of the cell, PM behaves as an insulator. However, certain ions, such as Na^+^ and K^+^ ions are pumped by the Na^+^-K^+^ ATPase which causes ion imbalance on either side of the membrane. This imbalance creates the resting transmembrane potential difference (resting ∆TMP). When cells are subjected to an electric field, the mobile charges (mainly ions) will move under the action of the electrophoretic force generated by the electric field, which will result in a further charging of the membrane: the so-called induced ∆TMP, superposing to the resting ∆TMP. When the net ∆TMP reaches a critical value, called permeabilization threshold, the properties of the membrane change: electroporation occurs, the membrane is electropermeabilized and it loses its insulating character. Depending on the amplitude of the field and the duration of the pulses, the electropermeabilization can be reversible or irreversible. In the case of the reversible electroporation, the increased permeability of the PM persists for a few minutes and afterwards the membrane returns to its original impermeable state.

Electric pulses of a typical duration of 100 microseconds and an electric field amplitude of the order of 1000 V/cm (μsPEF), have been widely used in many biotechnological or medical applications, notably for antitumor electrochemotherapy^4–6^, tumor ablation^7,8^, cell transfection *in vitro* ^3,9^ and even gene transfer *in vivo* ^10,11^. μsPEF have been used to permeabilize many cell types and allow internalization of non-permeant molecules such as ^51^Cr-EDTA^12^, bleomycin^13^, or plasmid DNA^10,14^ either *in vitro* and *in vivo*. Such pulses may also be used to achieve the fusion of adjacent cells brought in contact to each other^15^ or even to break the integrity of the cell membrane and cause cell death in the case of the achievement of irreversible electroporation^7,16^.

Since the early 2000s, electric pulses of a few nanoseconds to several hundreds of nanoseconds and field strength up to 300 kV/cm are used in biology: the so-called nanosecond pulsed electric fields (nsPEF). As the pulse duration in this type of pulses is below the PM charging time constant, effects at the PM level decrease and effects on intracellular membranes become detectable^17,18^. Unlike the μsPEF, these ultrashort pulses have already been shown to permeabilize both the PM and the organelles membranes^19^.

Small ions such as Ca^2+^ can be used to visualize the PM or the inner stores permeabilization through the use of fluorescent markers like Fluo-4, or Fura-2. Indeed, there is a large difference of Ca^2+^ concentration between the cytoplasm and the extracellular medium (respectively about 100 nM and 1.2–1.8 mM). The same large difference is present between the cytoplasm and the endoplasmic reticulum (ER) (respectively 100 nM and 0.05 to 1 mM). Therefore, when permeabilization of the PM or ER membranes occurs, a large number of molecules will enter the cytosol with gradient-driven very fast kinetics.

In the study here reported, Ca^2+^ was used as a marker of internal or external membrane electropermeabilization in two very different types of cells exposed to one single 100 μsPEF. Response curves of Chinese hamster lung fibroblast cells (DC-3F) and human adipose mesenchymal stem cells (haMSC) to different electric field amplitudes were achieved. Characterization of the response included the number of cells presenting a Ca^2+^ peak in media with and without Ca^2+^, as well as the mean amplitude of the Ca^2+^ peaks. Cell viability was also tested. The Ca^2+^ peaks detected in a medium without Ca^2+^ demonstrate that µsPEF can also permeabilize the inner membranes of the cells without causing a loss of cell viability. To our knowledge, this is the first experimental study, with cell viability investigation, that shows that “classical” μsPEF can permeabilize internal membranes of the cells. Furthermore, to explain the amplitude of the various thresholds found, a detailed analysis of the ER structure in the haMSC and DC-3F cells was also performed.

## Results

### HaMSC exposure to one 100 µs electric pulse

**Figure 1:**
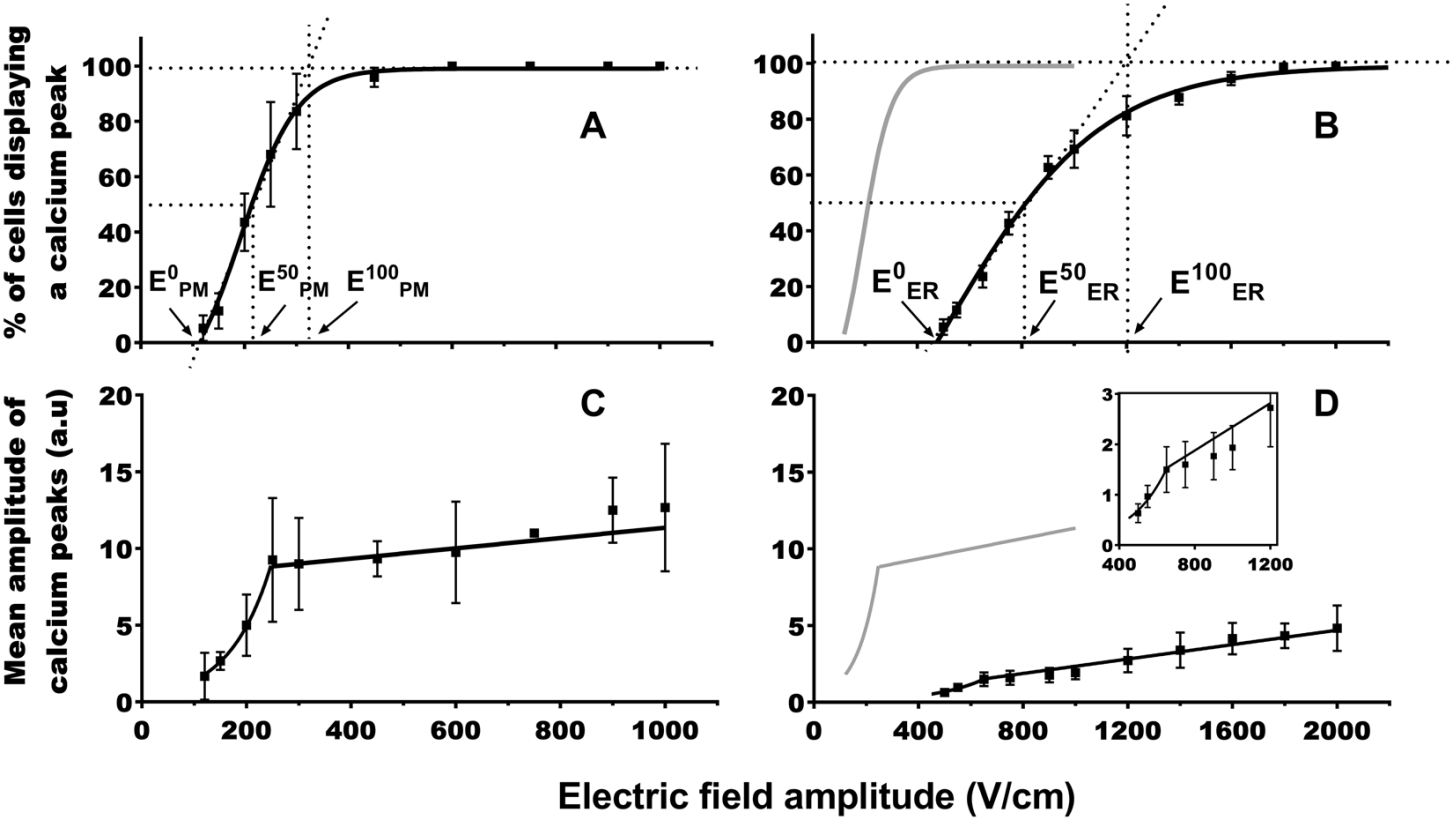
Response of the haMSC exposed to one pulse of 100 μs in DMEM (panels A and C) or in SMEM-EGTA (panels B and D). (**A**,**B**) Percentage of cells displaying calcium peaks. (C and D): Mean amplitude of the calcium peaks. E^0^, E^50^, and E^100^ are the values of the electric field amplitudes needed to respectively start to permeabilize the cells, and for the permeabilization of 50% and 100% of the cells. The gray curves in (**B**,**D**) represent the curves (**A**,**C**), respectively. The inset in (**D**) represent a magnification of the transition between the fast and the slow increase of the amplitude of the calcium peaks. (n = 3 to 7 independent experiments).

The haMSC mean radius was about 36 μm. In DMEM, cells displaying a pulse-induced Ca^2+^ peak could be detected at a field E^0^_PM_ (the electric field amplitude needed to permeabilize the plasma membrane in the first cells) as low as 120 V/cm (Fig. 1A). 50% of cells presented a calcium peak when pulse amplitude E^50^_PM_ (the electric field amplitude needed to permeabilize the plasma membrane in 50% of the cells) was about 210 V/cm and plateau was reached at E^100^_PM_ (the electric field amplitude needed to permeabilize the plasma membrane in 100% of the cells) at about 320 V/cm. E^50^_PM_ is simply the abscissa of the curve at the ordinate 50%. E^0^_PM_ and E^100^_PM_ are the abscissa of the intersections between the ordinates 0% and 100% and the tangent to the curve at E^50^_PM_. The curve representing the percentage of cells displaying a pulse-induced Ca^2+^ peak fits with a sigmoid. An exponential increase of the calcium peaks mean amplitude (among the cells responding to the pulse) was observed between 120 and 250 V/cm, followed by no further significant increase from 450 to 1000 V/cm (Fig. 1C). Traces of the typical pulse-induced Ca^2+^ peaks are provided in supplementary Fig. S1. No permeabilization of the PM was detected after a pulse of 300 V/cm using the classical PM permeabilization marker yo-pro-1 (Supplementary Fig. S2). With yo-pro-1, permeabilization was detected at 600 V/cm.

In SMEM-EGTA, there was no free Ca^2+^ in the medium surrounding the cells. At about 500 V/cm, only few cells displayed a Ca^2+^ peak (Fig. 1B). The number of cells presenting Ca^2+^ peaks increased with the field strength, to reach 100% at about 1200 V/cm (E^100^_ER_: the electric field amplitude needed to permeabilize the endothelial reticulum membrane in 100% of the cells). At 800 V/cm, 50% of cells responded to the pulse (E^50^_ER_: the electric field amplitude needed to permeabilize the endothelial reticulum membrane in 50% of the cells). The curve could fit with a truncated sigmoid, between 500 and 1600 V/cm. The mean amplitude of the Ca^2+^ peaks increased rather rapidly between 500 and 650 V/cm, and very slowly for higher field amplitudes (Fig. 1D). Mean value reached a maximum of 5 a.u. The curve fits with an exponential between 500 and 650 V/cm and with a straight line above 650 V/cm.

### DC-3Fcells exposure to one 100 µs electric pulse

**Figure 2:**
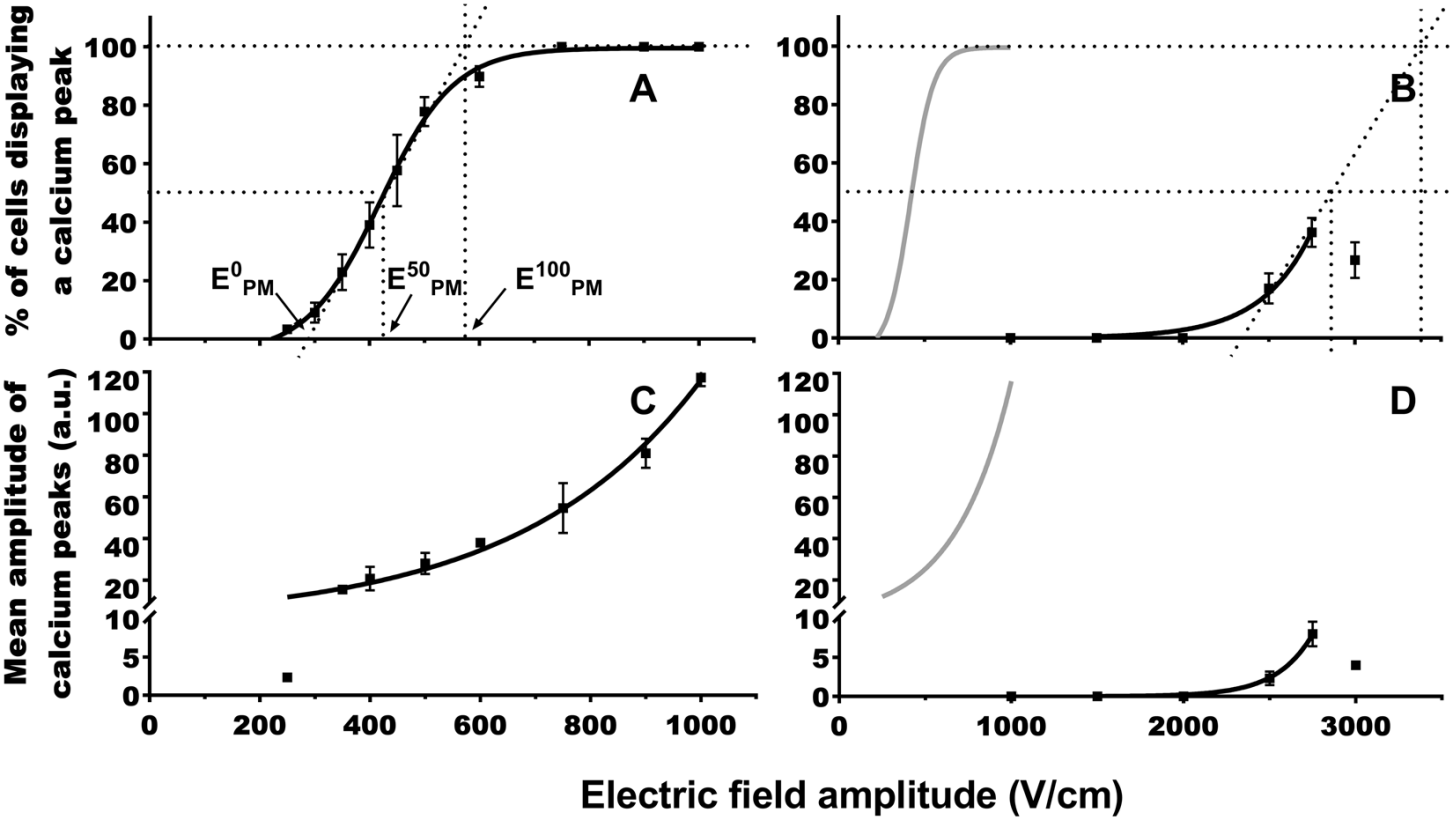
Response of the DC-3F cells exposed to one pulse of 100 μs in DMEM (panels A and C) or in SMEM-EGTA (panels B and D). (**A**,**B**) Percentage of cells displaying calcium peaks. (**C**,**D**) Mean amplitude of the calcium peaks. For E^0^, E^50^, and E^100^, refer to Fig. 1. The gray curves in (**B**,**D**) represent the curves (**A**,**C**), respectively. (n = 3 to 5 independent experiments).

The DC-3F cells mean radius was about 9 μm. In DMEM, 270 V/cm was the lowest field amplitude (E^0^_PM_) at which cytosolic Ca^2+^ peaks could be detected immediately after the pulse delivery (Fig. 2A). From 300 to 500 V/cm, the percentage of cells presenting a Ca^2+^ peak increased from 10 to 80%. At about 600 V/cm all the cells presented a Ca^2+^ peak (E^100^_PM_). 50% of cells responded to the pulse when amplitude E^50^_PM_ was about 430 V/cm. The curve fits with a sigmoid. The mean amplitude of the Ca^2+^ peaks increased monotonously between 200 and 1000 V/cm (Fig. 2C). The curve fits with an exponential. The mean amplitude of the peaks was higher than in the haMSC. Traces of the typical pulse-induced Ca^2+^ peaks are provided in supplementary Fig. S1. No PM permeabilization was detected after one pulse of 300 V/cm using yo-pro-1 (Supplementary Fig. S2). With yo-pro-1, permeabilization was detected at 600 V/cm.

In SMEM-EGTA, a high field strength (2500 V/cm) was needed to observe Ca^2+^ peaks in 20% DC-3F cells (Fig. 2B). The percentage of cells presenting a Ca^2+^ peak increased to 38% at 2750 V/cm and then decreased to 27% at 3000 V/cm. The curve between 2000 and 2750 V/cm could be fitted by an exponential. The curve presenting the mean amplitude of Ca^2+^ peaks (Fig. 2D) increased from 2 to 8 a.u. between 2500 to 2750 V/cm, and then decreased to 4 a.u. at 3000 V/cm.

### Loading of the cells with 15 µM fluo-4 AM or with calcein AM

**Figure 3:**
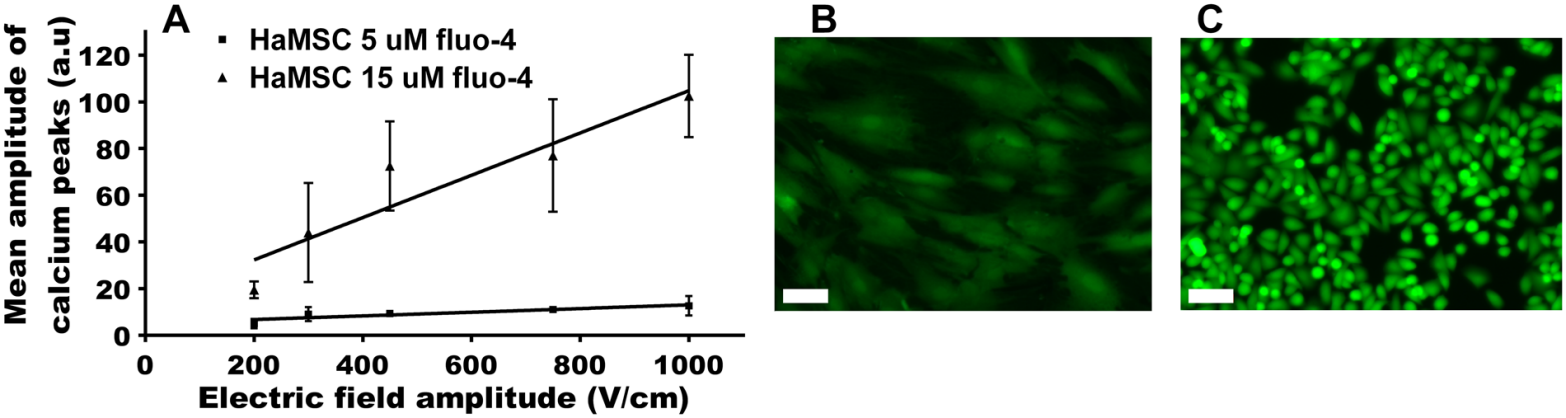
(**A**) Mean amplitude of the calcium peaks in haMSC after incubation of the cells in 5 or 15 µM fluo-4 AM (n = 3 independent experiments); (**B**,**C**) Calcein fluorescence in haMSC (panel B) and DC-3F (panel C) cells incubated in the presence of 5 µM calcein AM for 30 minutes at 37 °C. Scale bar 50 µm.

The saturation of the calcium peaks amplitude observed above 450 V/cm for MSC pulsed in presence of external calcium (Fig. 1C) could be due to a limited penetration of the external calcium or to a saturation of the fluo-4 inside the MSC. To test this hypothesis, we raised the external concentration of fluo-4-AM to 15 µM. This three times increase in fluo-4AM concentration did not modify the percentage of cells displaying a Ca^2+^ peak but caused a significant increase in the peaks mean amplitude. With 5 μM fluo-4-AM, Ca^2+^ peaks mean amplitude in haMSC was 9 and 13 a.u. for 300 and 1000 V/cm (Fig. 1C) while with 15 μM fluo-4-AM, it became respectively 45 and 105 a.u. (Fig. 3A). In the latter case, the curve fits also with a straight line between 300 and 1000 V/cm but with a steeper slope.

In DC-3F no such saturation was observed, probably because of a better accumulation of the fluo-4. To confirm that DC-3F tends to accumulate acetoxymethyl ester dyes better than the MSC, we compared calcein AM accumulation in MSC and DC-3F. For calcein, fluorescence in DC-3F cells was higher than in haMSC (Fig. 3B and C). The calcein-AM uses the same strategy as fluo-4-AM to enter the cell (that is the presence of the acetoxymethyl hydrophobic group) but once in the cell, its fluorescence does not depend on the Ca^2+^ presence.

### Inhibition of the Voltage-Operated Calcium Channels (VOCCs)

**Figure 4:**
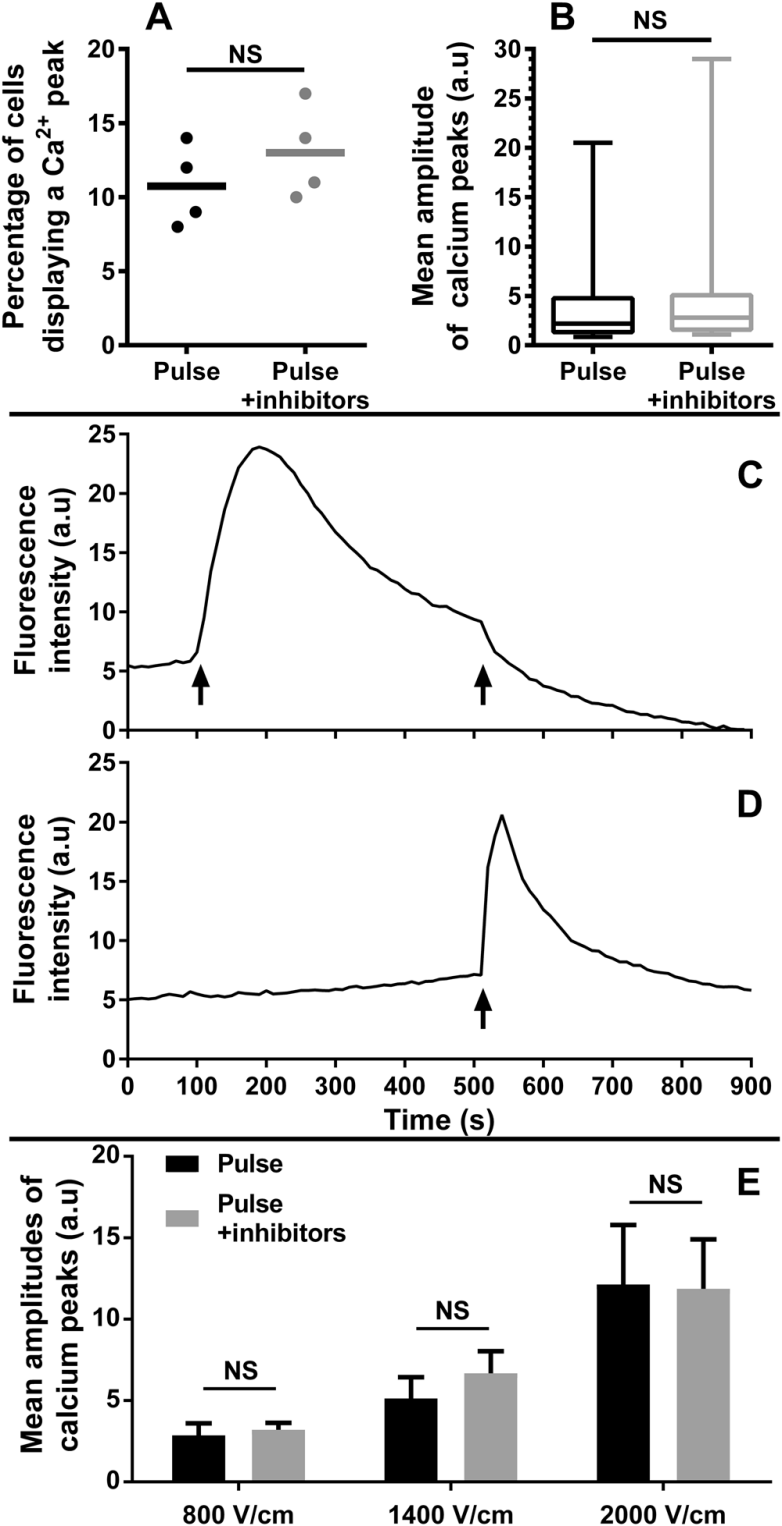
Origin of the calcium peaks. Cells were incubated with 10 μM of Fluo-4 AM and exposed to various inhibitors. (**A**,**B**) Response of haMSC in DMEM in the presence of VOCCs inhibitors. (**A**) percentage of cells presenting a Ca^2+^ peak (n = 4 independent experiments), the line represents the mean, p = 0.4 (Mann-Whitney test). (**B**) Mean amplitude of Ca^2+^ peaks (4 independent experiments with 39 cells for “Pulse” and 49 cells for “Pulse + inhibitors”), whiskers indicate 5th to 95th percentile range, p = 0.828 (Mann-Whitney test). Cells were exposed to one pulse of 150 V/cm (100 µs), in the presence or absence of 10 µM verapamil and 5 µM mibefradil. (**C**,**D**) Response of haMSC in SMEM-EGTA with or without thapsigargin addition. Arrow at 100 s: addition of 2 µM thapsigargin. arrows at 500 s: electric pulse of 2000 V/cm. The background fluctuations were corrected by subtracting the fluorescence of an area without cells for every time point. Graphs are representative of 3 independent experiments (**E**) haMSC electropulsed in a medium without calcium (SMEM-EGTA) mean amplitude of the calcium peaks in the presence or the absence of 50 µM of 2-APB, 50 µM of Dantrolene and 25 µM of Flecainide. Data are mean ± SD (n = 3 independent experiments). No significant effect of the inhibitors was observed (two-way ANOVA followed by Tukey multiple comparisons test).

HaMSC cells were exposed to one single 100 µs electric pulse in DMEM with or without verapamil and mibefradil, inhibitors respectively of L-type and T-type VOCCs^20,21^. Even at the lowest electric field amplitude (150 V/cm, resulting in about 10% of cells displaying an electro-induced Ca^2+^ peak), the use of VOCCs blockers did not affect the pulse-induced Ca^2+^ peaks (p = 0.4, Mann Whitney test) (Fig. 4 panels A and B and supplementary Fig. S3). Similarly, in DC-3F, even at 300 V/cm the use of VOCCs blockers did not affect the percentage of cells presenting a pulse-induced Ca^2+^ peaks (8.7% of DC-3F displayed an electro-induced Ca^2+^ peak without inhibitor compare to 9.5% with VOCCS inhibitors), nor the amplitude of these peaks (supplementary Fig. S4).

### Inhibition of Sarco/Endoplasmic Reticulum Calcium ATPase (SERCA)

After the addition of 2 µM of thapsigargin (a SERCA inhibitor)^22^ to haMSC in SMEM-EGTA medium, an increase of the cytosolic Ca^2+^ concentration was observed (Fig. 4 panels C and D), corresponding to the depletion of the ER Ca^2+^ store^23^. When a 2000 V/cm electric pulse (100 µs) was applied after the addition of thapsigargin, no electro-induced Ca^2+^ peak was observed. On the contrary, the electric pulse caused a little decrease in fluo-4 fluorescence. As previously observed in Fig. 1, when the same pulse (2000 V/cm) was applied without a prior addition of thapsigargin, it provoked an electro-induced Ca^2+^ peak.

### Inhibition of the inositol triphosphate and ryanodine receptors

HaMSC cells were incubated with or without 2-aminoethoxydiphenyl borate (2-APB), dantrolene sodium salt, and flecainide acetate salt, inhibitors respectively of inositol trisphosphate receptor (IP3R), ryanodine receptor (RyR) 1, 2 and RyR 3^24–26^. In all the cases, Ca^2+^ peaks mean amplitude increased with the field strength (Fig. 4 panel E and supplementary Fig. S5). No differences in the mean amplitude were observed between cells with or without inhibitors (p = 0.1384, 2-way ANOVA test), while there was a significant effect of the field strength applied according to the same test (p = 0.0035).

### Comparison of the endoplasmic reticulum of the two cell types

**Figure 5:**
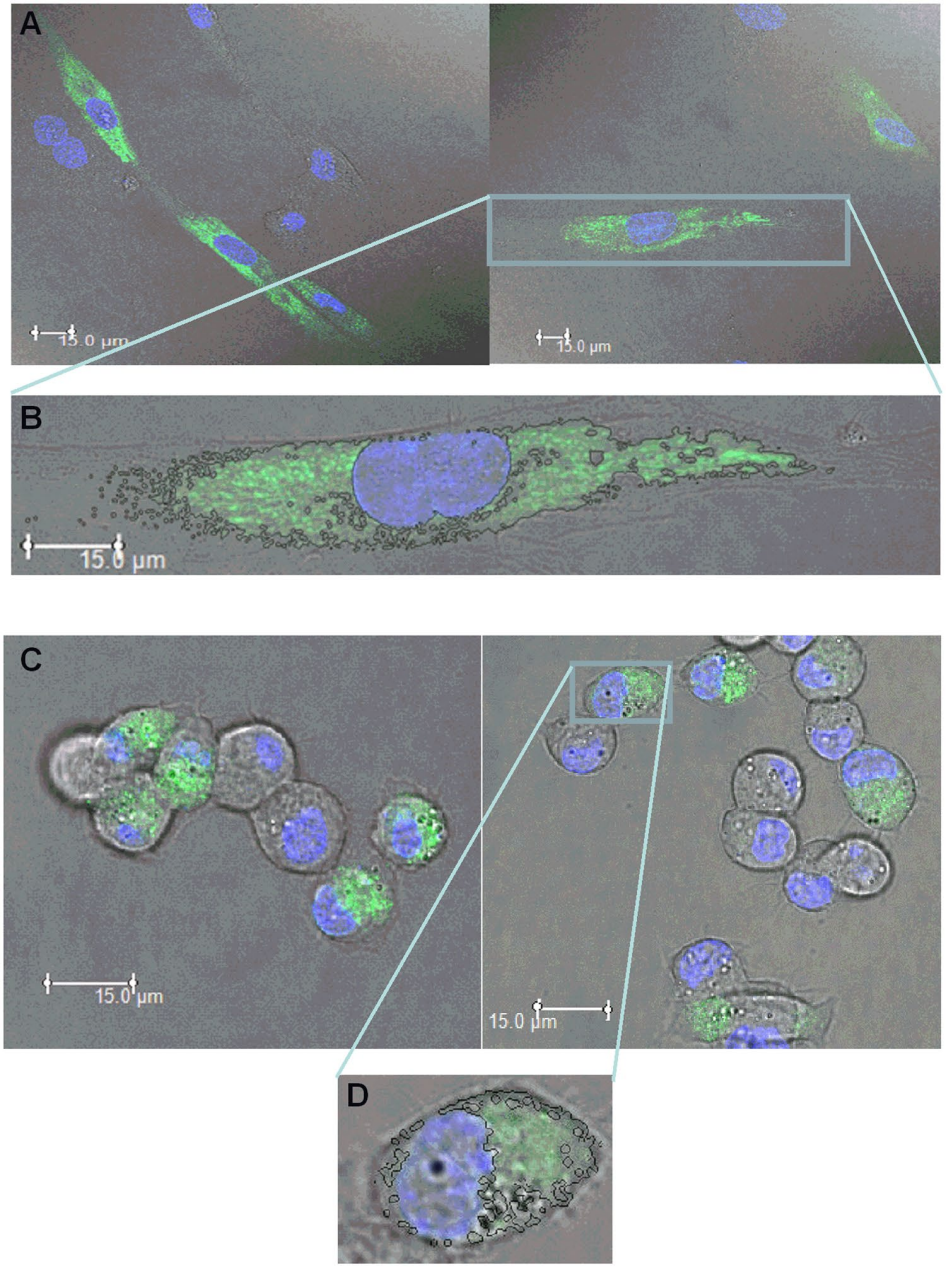
Endoplasmic reticulum of the 2 cell types labelled by the protein D1ER (×63). (**A**,**B**) haMSC; (**C**,**D**) DC-3F cells. The images were taken without zooming for the haMSC and using a ×2 zoom for the DC-3F cells (Nuclei were labelled with by Hoechst 33342). (**A**,**C**) Confocal pictures. (**B**,**D**) examples of ER identification.

The ER was decorated by the D1ER protein. In attached haMSC, nuclei were located at the center of the cells and the ER completely surrounded the nuclei, occupying a large volume in the cell. The ER 3D reconstruction allowed getting precise ER dimensions. The maximal diameter of the ER was approximately 75% of the haMSC diameter. In the attached DC-3F cells, nucleus was located in general at one side of the cell and the ER extended mainly in the other side of the cell. Maximal ER diameter did not surpass 45% of DC-3F cells diameter (Fig. 5).

### Cell viability after one 100 µs electric pulse

**Figure 6:**
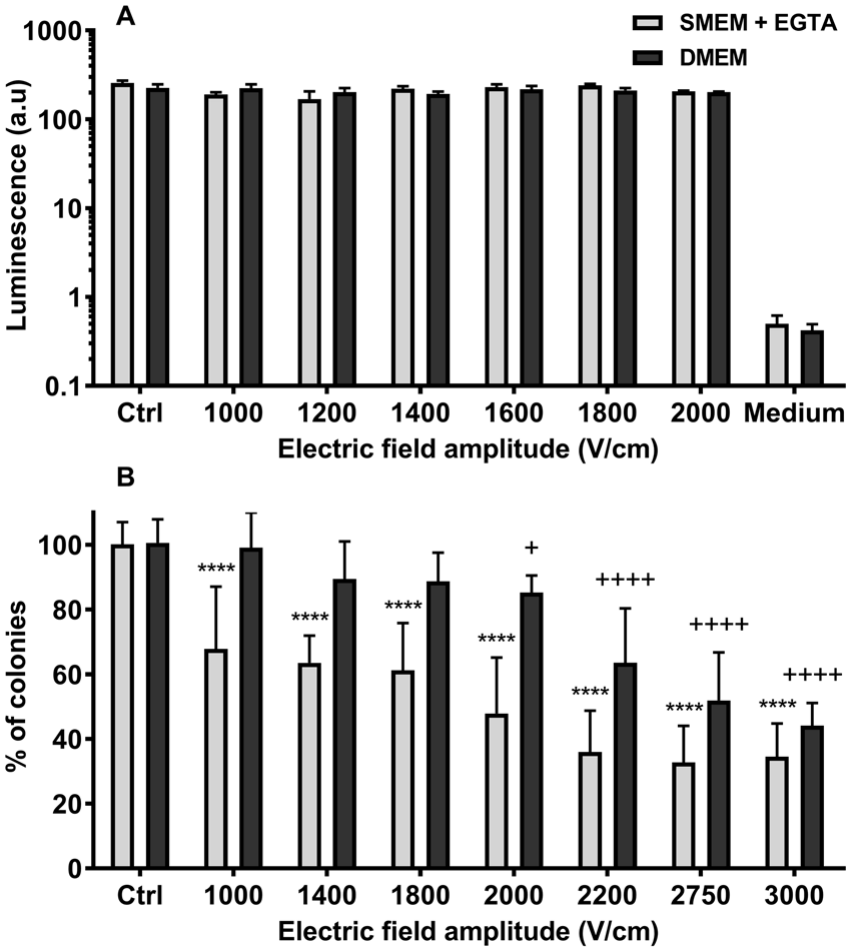
Cells viability after exposure to one 100 μs pulse in a medium with calcium (DMEM) or without calcium (SMEM-EGTA). (**A**) haMSC (n = 3 independent experiments). (**B**) DC-3F cells (n = 3 to 5 independent experiments in triplicates). Data are means ± SD. Statistical significance represented: p < 0.0001 (++++ and ****), p < 0.05 (+) are for comparison respectively to the DMEM and SMEM-EGTA controls (Ctrl) (two-way ANOVA followed by Dunnett’s multiple comparison test).

Up to 2000 V/cm there was no significant difference in cell mortality between haMSC unexposed (ctrl) or exposed to one pulse (Fig. 6A). haMSC viability was almost the same in the two media, with and without calcium.

In DMEM, one pulse of 1000 V/cm did not impact DC-3F cells viability (Fig. 6B). A little decrease in the apparent DC-3F cells viability, from 100 to about 87%, was observed between 1400 and 2000 V/cm. Above 2000 V/cm the number of colonies decreased with the field amplitude, with 60% of cells still making colonies at 2750 V/cm, and 40% at 3000 V/cm.

In SMEM-EGTA, a decrease of DC-3F cells viability to 60–65% was observed even at 1000 V/cm and until 1800 V/cm. At 2000 V/cm, only 50% of cells made colonies. This percentage decreased at 2200 V/cm to reach 30%, and no further decrease was found at higher field amplitudes.

### Formation of DC-3F polykaryons after one µsPEF in SMEM-EGTA

**Figure 7:**
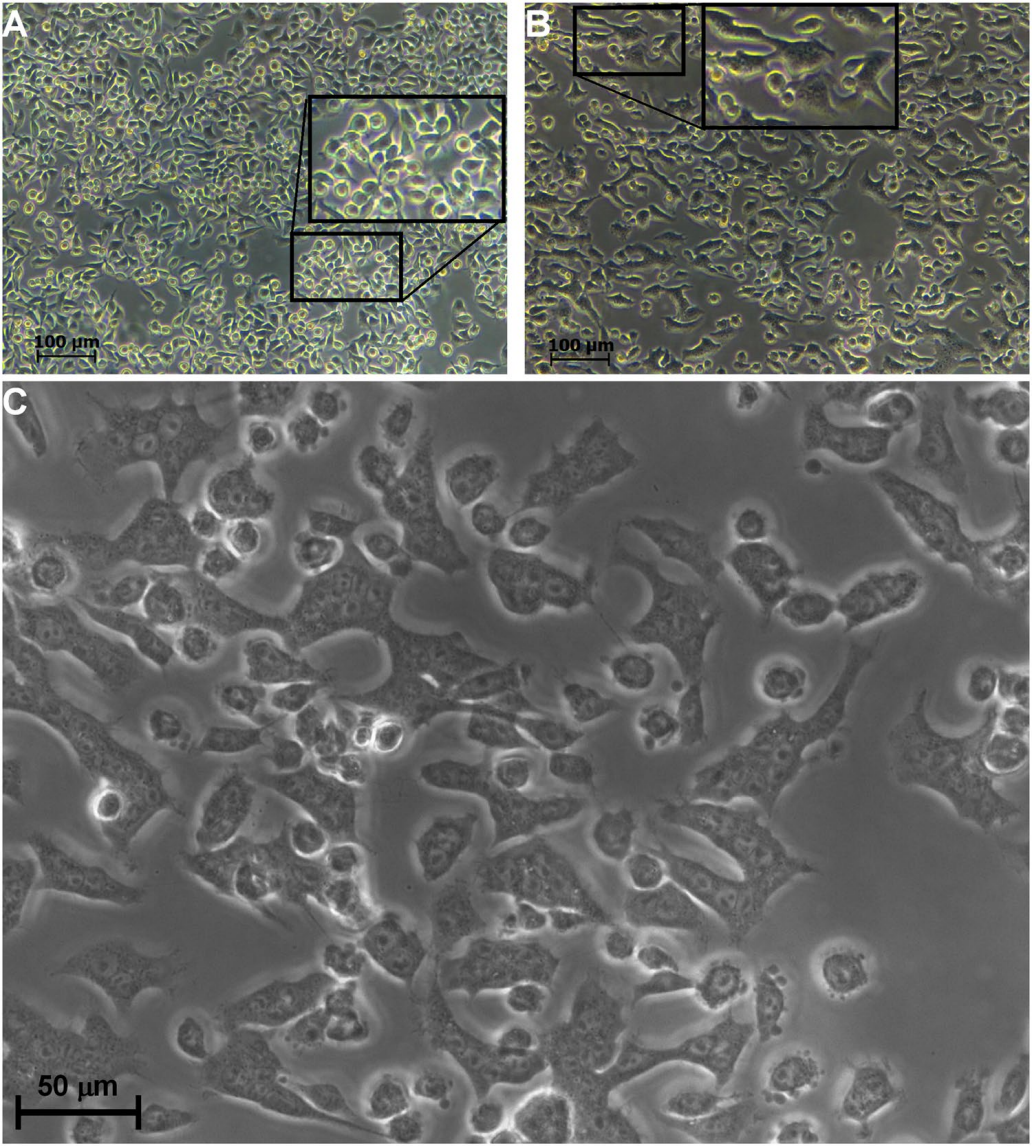
DC-3F cells electrofusion after one pulse in SMEM-EGTA. (**A**) control, (**B**,**C**) 20 min after a pulse of 2000 V/cm.

To analyze the causes of the apparent decrease of viability of the DC-3F cells treated in the SMEM-EGTA medium, cells were continuously monitored under the microscope for 20 min directly after the pulse delivery. Actually, many cells fused rapidly (Fig. 7): fusion events were detected even five min only after the pulse. The number of fusions could not be quantitatively assessed.

## Discussion

μsPEF are an effective tool used in many domains such as research, medicine and biotechnology, for antitumor electrochemotherapy, tumor ablation, cell transfection, etc. Due to their duration, which is long enough to cause transmembrane potential changes, μsPEF were normally supposed to solely interact with the PM and change its permeability properties if the field amplitude reached a certain value. If the investigators desire to permeabilize internal membranes, nsPEF (pulses of a few nanoseconds duration) are applied^27,28^. Indeed, using nsPEF, internal membranes such as mitochondrial^29^ or endosomal^30^ membranes were shown to be permeabilized. Thus, in the case of the μsPEF (classical duration: 100 µs), it is widely accepted that only the external membrane is affected by the electric pulses.

Only a couple of theoretical studies simulated the effect of the μsPEF on organelles^31,32^, and so far, no experimental study showed such an effect. Our quantitative study demonstrates for the first time that internal membranes can be permeabilized with a single 100 μs pulse, while preserving cell viability. The amplitude of the pulse strength is highly dependent on the cell type.

haMSC and DC-3F cells and two media, with and without calcium, were used here to study calcium peaks generation in cells exposed to one 100 μs pulse of field amplitudes ranging from 120 to 3000 V/cm. The DC-3F cells are a hamster cell line whereas the haMSC are human primary cells. Moreover, the DC-3F cells are small cells whereas the haMSC are large ones. The origin and size difference was important for us in order to have two models of cells and to study the interaction of the electric field with their PM and ER. One single electric pulse was delivered because we recently showed that pulses repetition frequency impacts the efficacy of the PM permeabilization when several pulses are delivered^33^. Thus, the real impact of electric pulses on different cells and/or organelles is easier to evaluate if a single pulse is delivered. In further studies, it will be necessary to analyze the impact of pulses repetition frequency on organelles electropermeabilization. To distinguish between PM permeabilization and internal organelles permeabilization, we used a medium with classical Ca^2+^ concentration (DMEM; 1.8 mM CaCl_2_) and a medium completely deprived of Ca^2+^ (SMEM-EGTA; not only the SMEM is prepared without Ca^2+^ ions, but moreover we added EGTA to complex any remaining trace of Ca^2+^ ions). In DMEM, a Ca^2+^ peak (detected by the increase in the cytosolic Fluo-4 Ca^2+^ marker fluorescence) is mainly the result of Ca^2+^ influx from the external medium, across the PM, but a contribution of Ca^2+^ release from inner stores cannot be excluded. On the contrary, in the SMEM-EGTA, any observed cytosolic Ca^2+^ peak must be due to Ca^2+^ released from the Ca^2+^ inner stores.

### Plasma membrane permeabilization

The comparison of the two curves achieved in the presence or absence of Ca^2+^ (Figs 1 and 2 panels A and B) demonstrates that, up to certain field amplitude, only extracellular Ca^2+^ contributes to the peak in a medium with Ca^2+^. Indeed, in haMSC and DC-3F cells, 100% of cells in DMEM displayed a Ca^2+^ peak respectively at 450 and 700 V/cm (Figs 1 and 2 panels A), electric field amplitudes at which no Ca^2+^ peak was detected in SMEM-EGTA (Figs 1 and 2 panels B).

This influx of extracellular calcium induced by the electric pulse could be the result of the electropermeabilization of the cell membrane or/and the activation of VOCCs. In the literature electropermeabilization is usually reported for transmembrane potentials of at least 200 mV^34^. But these values are based on the penetration of large fluorescent dyes such as propidium iodide which are much larger than a calcium ion. For small ions like calcium, electropermeabilization probably occurs at lower applied electric field. Indeed, several groups have reported that electropermeabilization to molecules of increasing sizes requires pulses of increasing electric field strengths^35–40^. However, activation of VOCCs occurs for an even lower membrane depolarization (30–50 mV)^41^. Moreover, it has been reported that nsPEF can trigger calcium influx via VOCCs^42^. We have thus exposed MSCs to the electric pulse in the presence of L-type and T-type VOCCs inhibitors. Even at the lowest electric field amplitude, the use of VOCCs blockers did not affect the pulse-induced Ca^2+^ peaks (Fig. 4 panels A and B and supplementary Fig. S3). Thus, VOCCs do not participate in the calcium peaks induced by the electric pulses, which is coherent with the fact that only 10–15% of MSCs express VOCCs^43,44^. For DC-3F, the pulse-induced Ca^2+^ peaks were not affected by the VOCCs blockers either (Supplementary Fig. S4). Therefore, in our experiments the external calcium is most likely entering the cytoplasm through the electropermeabilized membrane. Penetration of Ca^2+^ from the outside of the cell due to PM electropermeabilization has already been reported albeit with longer pulse duration or at higher electric field amplitudes^45,46^. Tumor treatment by penetration of large Ca^2+^ amounts after electroporation is even tested at the clinical level^45^.

### Endoplasmic reticulum permeabilization

The calcium peaks reported in haMSC and DC-3F cells in SMEM-EGTA (Figs 1 and 2 panels B) demonstrate that one single 100 µs pulse can mobilize Ca^2+^ from internal vesicles at field amplitudes higher than those permeabilizing the PM. Indeed, the total absence of Ca^2+^ in the external medium implies that the electro-induced Ca^2+^ peaks can only arise from the flow of Ca^2+^ from the internal stores to the cell cytosol. Since the ER is known to be the largest store of releasable Ca^2+^ in the cell^47^, these electro-induced Ca^2+^ peaks are probably due to the release of Ca^2+^ from the ER. To test this hypothesis, we depleted the ER calcium store through the inhibition of the SERCA (that pumps the Ca^2+^ from the cytosol to the ER) by thapsigargin (Fig. 4 panels C and D). When the thapsigargin was added to haMSC in SMEM-EGTA, an electric pulse of 2000 V/cm did not provoke an electro-induced Ca^2+^ peak. On the contrary, a small decrease in fluo-4 fluorescence was observed. Similar decreases of the fluo-4 fluorescence below the initial level were observed in Supplementary Fig. S1 only in absence of external calcium and for the highest electric field used. These decreases are probably due to the cytoplasmic calcium simply leaking out of the electropermeabilized cell. They may also be due to the high electric fields applied resulting in the penetration of the external EGTA in the cells and/or a partial leak of the fluo-4 through the highly permeabilized plasma membrane. However, when the same electric pulse was applied without a prior addition of thapsigargin, all the cells presented a Ca^2+^ peak. This demonstrates that the ER depletion by thapsigargin prevented the occurrence of the electro-induced Ca^2+^ peaks. Therefore, the major source of electro-induced Ca^2+^ spikes in SMEM-EGTA is the ER. This is coherent with the mathematical cell model of Esser *et al*.^31^ who showed that a single 40 µs electric pulse can cause transient electric perturbations of all organelle transmembrane voltages, preferably on the larger organelles such as the ER compared to smaller organelles such as the mitochondria. This is also identical to what has been reported with nsPEF where the ER was also the only appreciable source of intracellular calcium increase in response to nsPEF stimulation in a calcium free buffer^48^.

To assess if the Ca^2+^ peaks observed in SMEM-EGTA were the result of the ER membrane electropermeabilization or a release of Ca^2+^ through the IP3R and RyR due to cellular signaling, experiments with inhibitors of these channels were performed at three field strengths. The IP3R and RyR are the two channels from which Ca^2+^ is released from the ER: the IP3R plays an important role in the transduction of the external stimuli to specific intracellular Ca^2+^ signals^49^. Both RyR and IP3R are activated by a cytosolic increase in Ca^2+^concentration^50^. Even in the simultaneous presence of the three inhibitors, Ca^2+^ peaks always appeared in SMEM-EGTA (Fig. 4 panel E and Supplementary Fig. S5). The concentrations of inhibitors used in our experiments were sufficient to block the natural calcium oscillation of the haMSC in presence of external calcium, and even a 4-time increase of the inhibitors concentration did not affect the pulse induced Ca^2+^ peaks observed without external calcium (data not shown). All these results demonstrate that without external calcium, the electro-induced Ca^2+^ peaks are due to the release of calcium through the ER electropermeabilized membrane. Semenov *et al*.^48^ reported that with nsPEF, above a certain threshold, the calcium release from the electropermeabilized ER was biologically amplified via the calcium induced calcium release (CICR) positive feedback mechanism. In our case, the CICR is not activated without external calcium, even at 2000 V/cm, probably because the cytoplasmic concentration of calcium remains below the CICR activation threshold. However, in presence of external calcium, the cytoplasmic concentration of calcium reached is much higher and we cannot exclude an activation of the CICR.

Therefore, we bring the experimental evidence to the theoretical work of J. Weaver and colleagues^31,32^ who postulated that µsPEF can permeabilize cell organelles even though organelles radius is lower than cell radius and even if internal vesicles are shielded by the PM before the pulses delivery. Indeed, since the plasma membrane is permeabilized within the first microseconds of the electric pulse, the externally applied electric field can then penetrate inside the cells, modify the transmembrane voltage of organelles and even permeabilized them, provided that the electric field applied is high enough.

### Electroporation thresholds for PM and ER in haMSC and DC-3F cells

#### Electroporation thresholds for PM

The curves reporting the percentage of cells presenting a Ca^2+^ peak after one µsPEF in DMEM as a function of the field amplitude are sigmoids, with a shift to higher values of the field amplitude in the case of the DC-3F cells (approximately two times higher) (Figs 1 and 2 panel A).

It is important to note that these curves fit with sigmoids. Indeed, a sigmoid corresponds to the derivative of a Gaussian distribution, which is the type of distribution of the individual cell sizes within a cell population. This shift could be due to the difference in cell size, the haMSC mean radius being 4 times larger than the DC-3F cells one (respectively 36 and 9 μm). Indeed, H. Schwan^51^ and others later on^52^ analyzed the influence of the particle radius in the ∆TMP generated by an external field showing the reciprocal influence of the radius and the field strength. However, according to the Schwan equation ΔΨ = 3/2 E.R cos θ^51^ (where E: applied field strength; R: cell radius; θ: angle between field lines and a normal to the cell surface at the point of interest; ΔΨ: value of the electrically induced ∆TMP at this point of the cell surface), the electric field amplitudes between the two cell types should have differed by a factor of four, while they only differed by a factor of two. However, the Schwan equation is strictly valid for spherical cells in suspension. For attached cells the contribution of the cell size may not be linear. Moreover, other differences between the haMSC and DC-3F cells also contribute to the variation in the permeabilization threshold such as differences in cell shape, cell orientation and cell organization (Fig. 3 panels B and C)^53^.

All our data here were achieved using Ca^2+^ which is not a classical permeabilization marker. Therefore, a classical one, like the yo-pro-1 iodide^19,54^, was used to compare the PM permeabilization thresholds between the two markers (Supplementary Fig. S2). The results showed no cell permeabilization to yo-pro-1 at a field lower than 600 V/cm, whereas electropermeabilization to Ca^2+^ (in Ca^2+^-containing medium) was partly detectable at 120 and 270 V/cm for haMSC and DC-3F cells respectively. These results support the hypothesis that the electric field threshold needed to detect cell permeabilization is lower for a small molecule (like Ca^2+^) than for a large one. This is in agreement with the observations of several other groups^35–38^, and has also been observed with nanosecond electric pulses^39,40,55^.

#### Electroporation thresholds for ER

For the two cell types, higher electric field amplitudes were needed for the ER permeabilization than for the PM permeabilization (Figs 1 and 2 panels B versus panels A). For example, in haMSC, an about 4 times higher electric field amplitude was needed to observe the beginning of the permeabilization of the Ca^2+^ internal stores membranes (480 vs 120 V/cm) and about the same factor to obtain the Ca^2+^ peaks in 50% of the cells (800 vs 210 V/cm). In the case of nsPEF, the electric field threshold reported for ER permeabilization was also greater than the threshold for PM permeabilization^48^. This could be explained by the fact that the PM needs to be permeabilized first for the field lines to be able to penetrate inside the cell and permeabilize the cell organelles. Moreover, according to the Schwan equation and mathematical cell modeling^31,56^, higher field amplitudes are needed to permeabilize the organelles membranes due to the organelles smaller size.

Permeabilization of the ER starts to be detectable at 480 V/cm for MSCs (4 times higher than for their PM permeabilization) and above 2000 V/cm for DC-3F cells (7.4 times higher than for their PM permeabilization). The difference in ER size and distribution between the two cell types could contribute to this discrepancy. Indeed, while haMSC ER distributes in a large fraction of the cell and completely surrounds the nucleus (Fig. 5), DC-3F cells ER was found to be localized in a rather small part of the cell at one side of the nucleus. Moreover, using a mathematical cell model, it has been demonstrated that microsecond electric pulses cause a larger change in the organelle transmembrane voltage for larger organelles^31,56^.

### Mean amplitude of the cytosolic calcium peaks: comparison between the two media and the two cell types

The mean amplitude of the calcium peaks in the two cell types was higher in a medium with Ca^2+^ than in a medium without Ca^2+^. The flux of external Ca^2+^ across the membrane is thus higher than the Ca^2+^ release from the ER. The mean concentration of the Ca^2+^ in the cytosol varies between 100 and 400 nM^57,58^. The Ca^2+^ concentration in DMEM is 1.8 mM, near the one found in the body extracellular medium (around 2 mM^59,60^), (the free Ca^2+^ concentration in serum is between 1.2 and 1.5 mM (4.8 and 5.9 mg/dl)^61^) whereas it varies between 50 μM and 1 mM in the ER^62,63^. Considering these concentrations and the fact that the total quantity of Ca^2+^ in the DMEM (the extracellular medium) is larger than the quantity of Ca^2+^ stored in the vesicles, the different amplitude of the Ca^2+^ peaks can be easily understood. Furthermore, the PM is more exposed to the fields than the ER membranes, which are encaged inside the cell. The electric field lines have to interact first with the PM, thus creating pores, and then, enter the cell inside and interact with the ER membrane. Hence the PM permeabilized areas should be greater in number or larger in size than those created in the ER.

In a medium with Ca^2+^, an exponential increase of the calcium peaks mean amplitude was followed by a saturation in the case of haMSC. The second part of the haMSC response was not due to a limited access of Ca^2+^ ions but to a limitation in the fluo-4 cytosolic accumulation as demonstrated by using higher external concentrations of fluo-4-AM (Fig. 3). A three times increase in fluo-4AM concentration did not modify the percentage of cells displaying a Ca^2+^ peak but caused a significant increase in the peaks mean amplitude. We did not observe the same saturation with DC-3F cells probably because they have a larger capacity to accumulate acetoxymethyl ester dyes (possibly due to a higher esterase activity, or to a higher rate of internalization). Indeed, calcein-AM fluorescence in DC-3F cells was also higher than in haMSC (calcein-AM fluorescence is independent of the Ca^2+^ presence).

In a medium without Ca^2+^, both the percentage of DC-3F cells presenting calcium peaks and the mean amplitude of theses peaks reached a maximum at 2750 V/cm then decreased at 3000 V/cm. This decrease might be attributed to specific effects observed only at the very high electric fields such as a denaturation of the fluo-4, penetration of external EGTA in the cells and/or the partial leak of fluo-4 through the highly permeabilized plasma membrane.

### Cell Viability under the pulse conditions

Since massive Ca^2+^ entry into cell cytoplasm is involved in several modes of cell death^64,65^, it was expected that cell viability would be lower when cells were pulsed in the presence of Ca^2+^ or eventually that the viability would be the same in the 2 media. Actually, the results with DC-3F cells were the opposite since at all the field amplitudes, their viability in SMEM-EGTA was apparently lower than in DMEM. haMSC viability was not significantly affected after the delivery of one 100 µs pulse in the presence or absence of calcium. Even at 2000 V/cm (where the internal Ca^2+^ stores were permeabilized in all the haMSC cells), the percentage of haMSC viability was about 85%.

To understand these results, microscopic observations of the DC-3F cells were performed for 20 min directly after the pulses. In SMEM-EGTA, the videos showed rapid movements of the cells and polykaryons began to be observed at short times (even 5 minutes) after the pulse delivery (Fig. 7). When cells were pulsed in DMEM, no cell fusion was observed within the first 20 minutes. The generation of polykaryons in SMEM-EGTA could explain, in part, the decrease in the number of colonies (that is the number of cells able to still replicate for several generations), and thus the apparent loss of viability, which was actually due to the cells electrofusion. The role of Ca^2+^ in the cell fusion procedure is controversial. Whereas some studies evocate a positive role in enhancing cell fusion^66^, another shows an inhibiting one^67^. Actually, fusion is a cell type dependent event, maybe related to differences in the extracellular matrix of the cells^68^ the fusogeneicity of the DC-3F cells in SMEM-EGTA was not found with the haMSC.

## Conclusion

The present study shows, for the first time, the electropermeabilization of the ER membrane by μsPEF, experimentally, and with cell viability assessments. It also demonstrates that one single micropulse with a low field amplitude is sufficient to permeabilize the cells PM to Ca^2+^ ions, a small size permeability marker. The importance of the ER size, distribution and architecture in the field amplitude needed to permeabilize the ER was revealed. This study brings the experimental evidence to previous simulation studies on the interaction of μsPEF with the internal membranes and establishes thresholds of permeabilization between PM and ER membrane, which are highly dependent on the cell type. It is also possible to conclude that the use of μsPEF is an efficient tool to modulate calcium concentration, through the interaction with the cell or the ER membranes. Since the Ca^2+^ signalization in the cell always implicate the ER, and since the Ca^2+^ is implicated in key cellular functions, the ER permeabilization could be an effective tool to study the role of Ca^2+^ in such functions and cellular physiology without a chemical stimulation and in a receptor-independent manner. This study demonstrates that, in some cases, µsPEF can be used to permeabilize internal membranes instead of nsPEF, with the interest that the µsPEF generation is cheaper, easier to control and more accessible to a large number of teams than the nsPEF technology.

## Materials and Methods

### Cells and cell culture conditions

HaMSC (Human adipose-derived mesenchymal stem cells), isolated from lipoaspirates (a plastic surgery waste) of individuals that volunteered and gave informed and written consent for the use of the lipoaspirates, were grown in DMEM (Dulbecco’s Modified Eagle Medium). DC-3F cells (Chinese hamster lung fibroblast cells) were grown in MEM (Minimum Essential Medium). Both media were supplemented with 10% fetal bovine serum, 100 U/mL penicillin and 100 mg/mL streptomycin. The cell culture chemicals were purchased from Fischer Scientific (Parc d’innovation Illkirch, France). Cells were propagated at 37 °C in a humidified 5% CO_2_ atmosphere.

HaMSC were passed every 3–4 days (one passage corresponding to one doubling time of the population). The multipotency capabilities of the cells were assessed by submitting them to differentiation conditions as previously reported by our group in André *et al*.^14^. DC-3F were routinely passed every 2 days.

Electric pulses delivery and calcium monitoring were performed under the microscope in DMEM or in SMEM-EGTA (Suspension Minimal Essential Medium supplemented with 2 mM final concentration of ethylene glycol tetraacetic acid *–* a calcium chelator). DMEM contained 1.8 mM CaCl_2_ whereas SMEM did not contain CaCl_2_.

### Cell staining

Cells were seeded in 24 well plates at a density of 5.10^4^ cells/cm^2^ (DC-3F cells) or 20.10^3^ cells/cm^2^ (haMSC) one day before the experiments. In order to visualize the effect of the μsPEF on living cells, the cells were incubated with 5 μM of Fluo-4 AM (Fischer Scientific), a fluorescent Ca^2+^ marker, for 30 min in a humidified 5% CO_2_ atmosphere at 37 °C in complete DMEM (haMSC) or complete MEM (DC-3F). To easily localize the cells, the incubation buffer also contained 375 nM of the nuclear fluorescent dye Hoechst 33342 (Fischer Scientific). After incubation, the wells were washed three times with PBS (Phosphate Buffered Saline) and then 500 μl of either DMEM or SMEM-EGTA were added.

### Inhibition of calcium channels and receptors

In order to inhibit the Voltage-Operated Calcium Channels (VOCCs), the cells were incubated for 30 min with 10 µM verapamil (L-type VOCC inhibitor) and 5 µM of mibefradil (T-type VOCC inhibitor) in a final volume of 500 μl of DMEM, in addition to Fluo-4 AM and Hoechst 33342. Then, the incubation medium was removed, the cells were washed 3 times with PBS and fresh DMEM medium containing the same concentration of the inhibitors was added to the cells.

In experiments devoted to inhibit the inositol trisphosphate receptors (IP3R) and ryanodine receptors (RyR), 50 μM of 2-aminoethoxydiphenyl borate (2-APB – an IP3R inhibitor), 50 μM of dantrolene sodium salt (a RyR1 and 3 inhibitor) and 25 μM flecainide acetate salt (a RyR2 inhibitor) were added to a final volume of 500 μl of SMEM-EGTA (in addition to Fluo-4 AM and Hoechst 33342) and incubated with the cells for 30 minutes. Then, the incubation medium was removed, the cells were washed 3 times with PBS and fresh SMEM-EGTA medium containing the same concentration of the inhibitors was added to the cells.

In order to empty the endoplasmic reticulum from calcium, 2 µM (final concentration) of thapsigargin, an inhibitor of the (SERCA) were directly added to the cells in SMEM-EGTA during the microscopic observation. The cells remained with 2 µM of thapsigargin for the rest of the microscopic observation. All drugs were purchased from Sigma Aldrich (St Quentin Fallavier, France).

In experiments devoted to test the intracellular loading of the acetoxymethyl ester form of the fluorophores, haMSC and DC-3f cells were loaded with a final concentration of 5 µM of calcein AM for 30 minutes, before observation using an epifluorescence microscope.

### Microsecond pulse generator and electrodes

μsPEF were generated by a Cliniporator^TM^ (Igea, Carpi, Italy). For the treatment of the cells under the microscope, the pulse generator was connected to two parallel stainless steel rods of 1.2 mm diameter used as electrodes. They were shaped to enter a 24 plate well and touch the bottom of the dish. The distance between the electrodes was always 5 mm, except when very high fields (>2000 V/cm) were applied. In this latter case the distance was 2 mm between the electrodes. The whole system was set under a Zeiss Axiovert S100 epifluorescence inverted microscope. One single micropulse of 100 μs was delivered in all the experiments.

For cell viability assessment, we designed a new model of electrodes. In this system, a thick cover of 10 cm^2^ was designed to fit in a Petri dish of the same dimensions. This cover contained 2 slots in which 2 plate electrodes of 2 mm thickness and 2 cm length could fit in: the electrodes were slipped into the slots until they touched the bottom of the Petri dish.

### Image analysis

Images of the cells were taken every 10 s for 10 to 20 min with a Zeiss AxioCam Hrc camera controlled by the Axio Vision 4.6 software (Carl Zeiss, Germany). The pulses were always delivered after at least 2 minutes of recording and 2 seconds before the next image. The excitation and emission wavelengths used for Fluo-4 were 496 nm and 515 nm respectively. The nuclear dye Hoechst 33342 (λex = 350 nm, λem = 461 nm) was used to track the cells during the videomicroscopy recording. There was no interference between Fluo-4 and Hoechst 33342 fluorescence because their emission wavelengths are separated enough. In this way, nuclei were recognized and the individual cells tracked using the Cell Profiler (version 2.0) software (Broad Institute, Cambridge, USA), allowing the automatic measurement of the fluorescence intensity signal of each cell on every image. Curves were plotted with a MATLAB program (version 7.8.0). All the observations were done at room temperature. The minimum opening time of the shutter for the fluorescent light was about 500 ms. To decrease the light energy applied on the cells, a 90% density Filter NE110B (Thorlabs, Maisons-Lafitte, France) was used.

### pcDNA-D1ER and endoplasmic reticulum imaging

pcDNA-D1ER was a gift from Amy Palmer & Roger Tsien (Addgene plasmid # 36325). This plasmid is coding for an endoplasmic reticulum (ER) marker fluorescing in green (535 nm)^69^. Cells were transfected with pcDNA-D1ER using 8 electric pulses of 100 μs and 1500 V/cm or 1200 V/cm (for the haMSC and DC-3F cells respectively) delivered at a repetition frequency of 1 Hz by the Cliniporator^TM^ (Igea, Carpi, Italy). We used 50 μg or 20 μg plasmid (for the haMSC and DC-3F cells respectively) in 100 μl, for 5.10^5^ cells in a cuvette of 1mm distance between the electrodes. D1ER is retained in the ER lumen through its C-terminal KDEL (lysine, aspartic acid, glutamic acid, leucine) sequence^70^.

A confocal microscope Leica TCS SPE with an objective HC PL APO CS2 63x, 1.30 NA oil and the LAS AF software version 3.3 (Leica, Germany) was used to visualize the endoplasmic reticulum, labelled by the ER marker. Excitation of the marker was done at 480 nm and emission captured at 535 nm. Cells nuclei were marked with Hoechst 33342 (excitation and emission at 405 and 486 nm respectively). The images were taken without electronic zooming for the haMSC and using a × 2 zoom for the DC-3F cells. 23 slices were taken each time to produce stacks of ER images with a Z distance of 0.8 µm between them.

The area of ER has been identified and reconstructed on the basis of the green fluorescence by using a custom semi-automated MATLAB^TM^ routine, obtained by a modification of the procedure of Joensuu *et al*.^71^. For each cell, the algorithm compiles all the stacks, taking only into account the green channel. The background noise was set to the 99.9% value of the areas without cells. This background was then subtracted to all the pixels of the images. Then, the threshold to identify the pixels belonging to the ER in the transfected cells was determined by considering the pixel value reflecting 95% of the fluorescence values of the non-transfected cells (called for simplicity negative cells). After the threshold identification, a clusterization of the images was carried out. Finally, on this binary matrix, extraction of the edges was performed resulting in the identification of the ER boundaries in the images. For all the slices at different depths, this procedure for ER identification was repeated. Then, each area was extruded for a thickness corresponded to the step of the microscopy procedure and finally elaborated with isosurface function in MATLABTM (v 2016) for the extraction of the 3D ER model as illustrated in supplementary Fig. S7.

### Microscopic observations of the DC-3F cells

In experiments devoted to detect cell fusion events in the electric pulse treated DC-3F cells, the Petri dish was transferred after the pulse delivery in a chamber set on the microscope stage to maintain the cells at 37 °C and 5% CO2, and a video of the cells was recorded for 20 min, with an image taken every 15 s.

### Cell viability assessment

Cells were seeded in 10 cm^2^ Petri dishes containing one 12 × 32 mm cover slide, at a density of 10^5^ cells/cm^2^ (DC-3F) or 20.10^3^ cells/cm^2^ (haMSC) one day prior to the experiments. After one day, complete medium was removed, cells were washed with PBS, and 1 ml of treatment medium (SMEM-EGTA, without Ca^2+^, or DMEM, with Ca^2+^) was added to the cells. The electrodes were placed and cells were pulsed (or sham exposed, for the non-pulsed controls). After the pulse, the Petri dishes were transferred into the incubator for 10 (DC-3F) or 20 minutes (haMSC). The placement of the electrodes causes the loss of cells beneath the electrodes. Interestingly, this allows recognizing the area between the electrodes, even after the removal of the electrodes. The cells outside of the electrodes were then scratched off the cover slides, under a sterile hood. Then, the cover slides were put in a cell culture incubator for 10 min in fresh complete MEM (DC-3F) or 2 hours in fresh complete DMEM (haMSC). The procedure is illustrated in Supplementary Fig. S6. The use of short incubation times (two times 10 min) in the case of the DC-3F cells was mandatory to prevent difficulty in cell trypsinization.

For DC-3F cells, viability was then assessed using the following clonogenic assay. One or two sham exposed coverslips were trypsinized and counted using a TC20 automated cell counter (Biorad). All the other coverslips (sham exposed and pulsed samples) were then trypsinized and diluted in complete medium at a final concentration of 1000 cells/8 ml (based on the number of cells previously counted in the one or two sham exposed coverslips). For each coverslip, cells were then distributed in 3 wells of a 6 wells plate (250 cells in 2 ml per well, in triplicate). The surviving cells formed colonies which were counted after 5 days.

Because haMSC do not form colonies, the assessment of haMSC viability could not be performed using the precise clonogenic assay test like in the case of the DC-3F cells. Therefore, pulsed haMSC cells were trypsinized, centrifuged, resuspended in 300 μl of fresh medium, and distributed in 2 wells of an opaque-walled 96 multiwell plate. After 24 hours in the incubator, 150 μl of Cell-Titer Glo Reagent (Promega) were added to each well according to manufacturer’s protocol. This caused cell lysis and generation of a luminescence signal proportional to the amount of ATP. The amount of ATP was directly proportional to the number of cells present in culture^72^. The luminescence was read on a GloMax luminometer (Promega).

### Calculation of the mean diameter of the cells and of the endoplasmic reticulum

50 cells from each cell type were chosen randomly in photos taken under the Zeiss Axiovert microscope. The larger diameter and its perpendicular diameter were measured, then the mean of the two was calculated for each cell (mean diameter). Then, the mean diameter for each cell type was calculated by averaging the mean diameters of 50 cells. The ER mean diameter for each cell type was measured in the same manner. Since the cells are randomly orientated in the electric field, the diameter of the cell in the electric field direction (apparent diameter) is, on the average, the mean diameter of the cells.

### Statistical analysis

All the experiments were done at least 3 times. Data are presented as means and standard deviations (except where indicated). To compare the effect of the VOCCs inhibitors, Mann-Whitney test was used. For the experiments using haMSC in a medium without calcium and with inhibitors of calcium channels, two-way ANOVA followed by Tukey multiple comparisons test was used. To compare the results of DC-3F viability, two-way ANOVA followed by Dunnett’s multiple comparison test was used.

## Electronic supplementary material

Supplementary Figures
