# Supplementary material for: Electropermeabilization of Inner and Outer Cell Membranes with Microsecond Pulsed Electric Fields: Quantitative Study with Calcium Ions
Source: Sci Rep. 2017 Oct 12;7:13079. doi: 10.1038/s41598-017-12960-w (PMC5638809; doi:10.1038/s41598-017-12960-w)
Supplement: Supplementary file 1 — Supplementary Figures [file 41598_2017_12960_MOESM1_ESM.pdf]

Electropermeabilization of Inner and Outer Cell Membranes with Microsecond Pulsed Electric Fields: Quantitative Study with Calcium Ions

Hanna Hanna [1], Agnese Denzi [2], Micaela Liberti [2], Franck M. Andre [1], Lluis M. Mir [1*]

[1] Vectorology and Anticancer Therapies, UMR 8203, CNRS, Univ. Paris-Sud, Gustave Roussy, Université Paris-Saclay, 94 805 Villejuif, France.

[2] Department of Information Engineering, Electronics and Telecommunication (DIET), University of Rome "La Sapienza," Rome, 00184, Italy

[*] To whom correspondence should be addressed.

E-mail address: luis.mir@gustaveroussy.fr

Tel. : +33 1 42 11 47 92

Laboratoire de Vectorologie et Thérapeutiques Anticancéreuses

UMR 8203 CNRS Univ Paris-Sud

Gustave Roussy

114 rue Edouard Vaillant

94805 VILLEJUIF Cédex France

Supplementary figures:

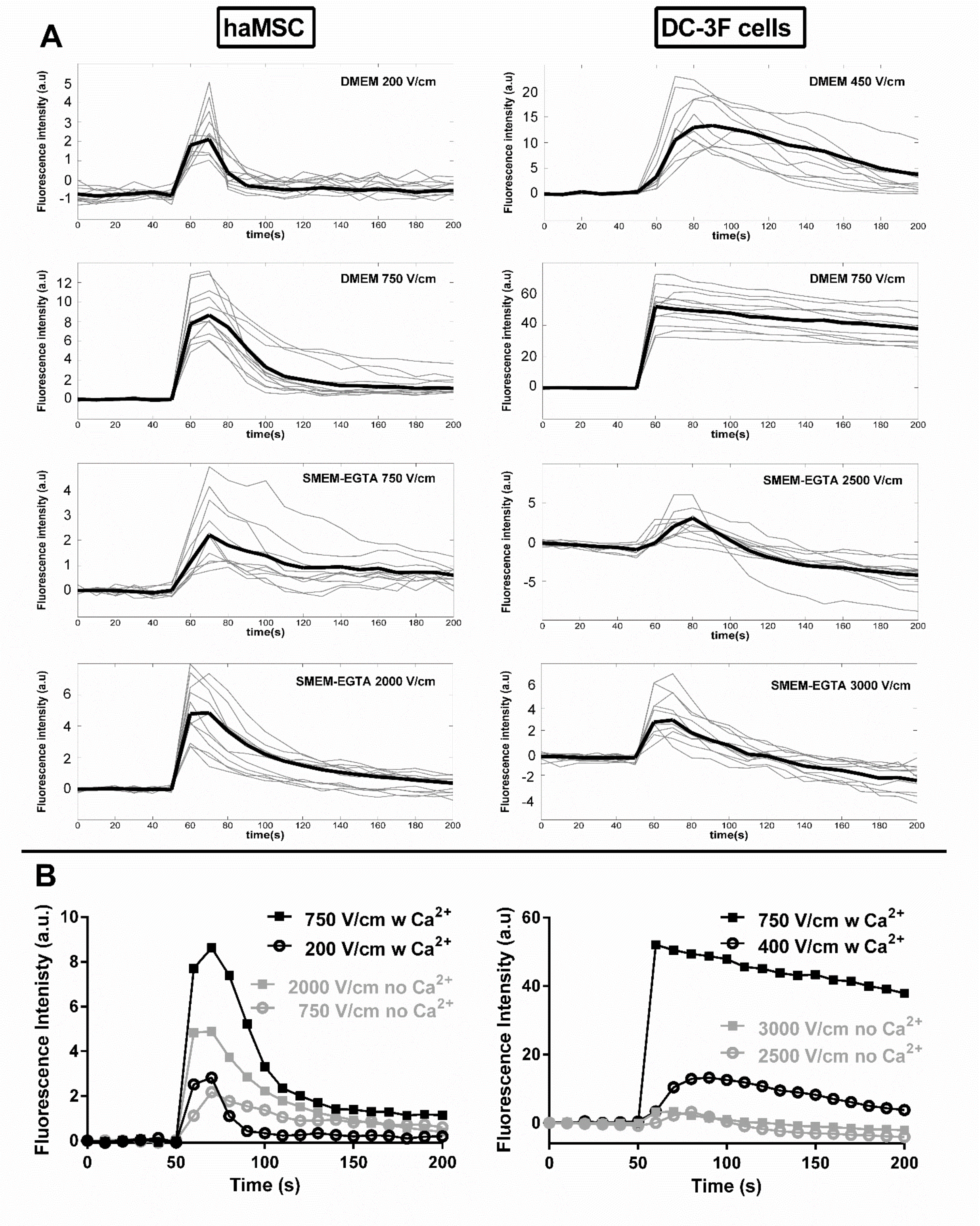

Figure S1. Typical traces of the pulse-induced Ca^{2+} peaks for the haMSC and the DC-3F cells in a medium with (DMEM) or without Ca^{2+} (SMEM-EGTA) after incubation of the cells in 5 µM fluo-4 AM. A: Shown are representative traces of the response in selected individual cells from the same experiment (gray lines, 10 cells per plot) and the average (thick black lines) of all the traces, not only of those displayed (3 to 7 independent experiments were performed in total). B: The averaged traces from A plotted on the same scale for better comparison.

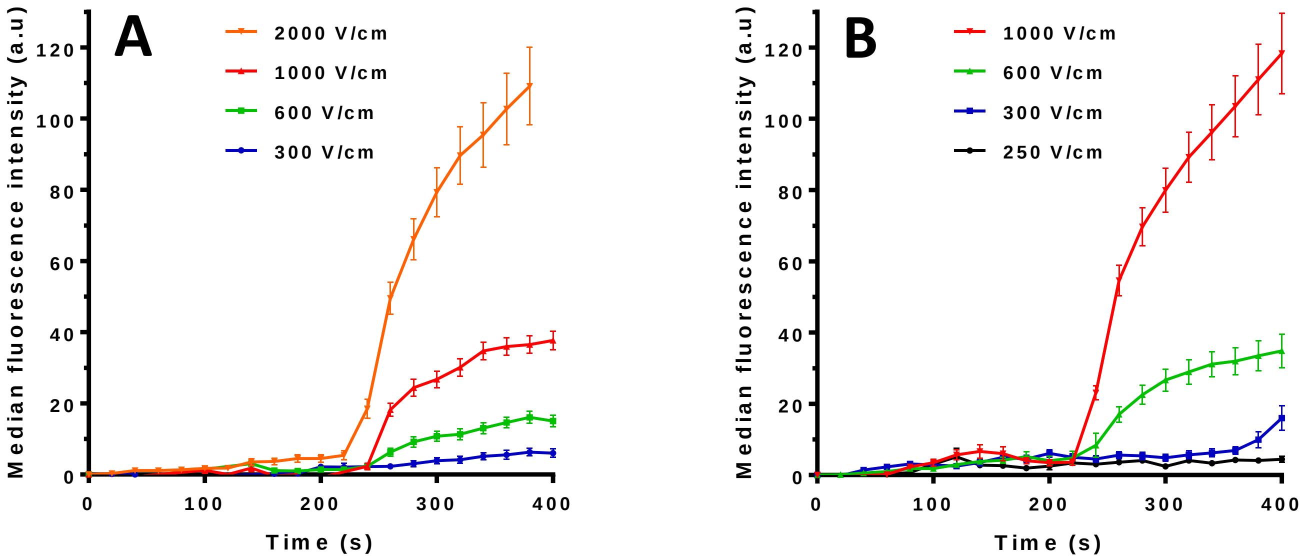

Figure S2. Evolution of the median fluorescence intensity of the cells electroporated in the presence of 5 μM yo-pro-1 iodide as a function of the pulse electric field amplitude. (*A*) about 70 haMSC were monitored for each electric field amplitude in 2 independent experiments. (*B*) About 140 DC-3F cells were monitored for each electric field amplitude in 2 independent experiments. Error bars represent the 95% confidence interval of the median fluorescence intensity. Photos were taken every 20s. Pulse was delivered at t=230 seconds. The initial auto fluorescence of the cells at time 0 was subtracted from all the measurements.

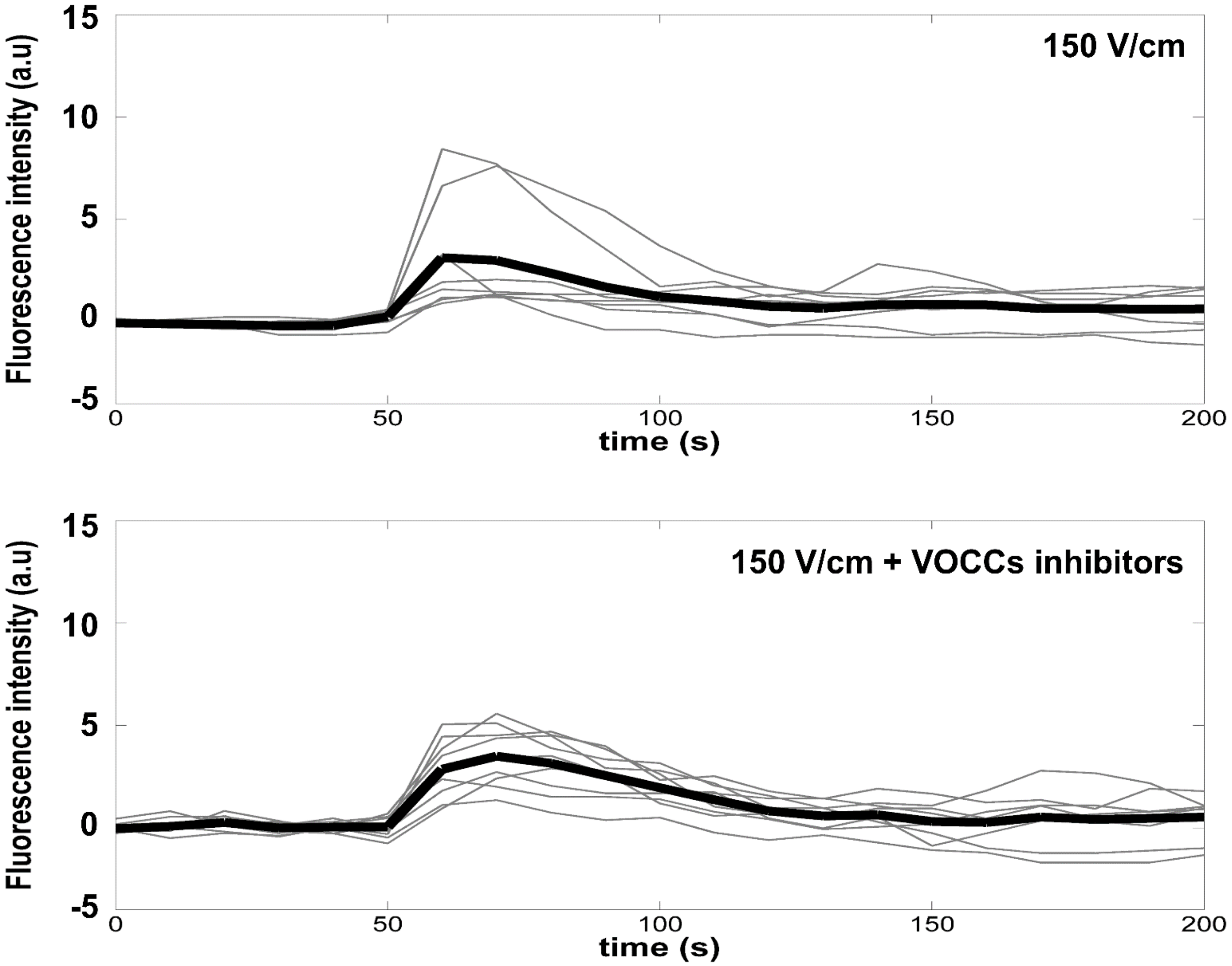

Figure S3. Typical traces of pulse-induced Ca2+ peaks for the haMSC in DMEM with or without VOCCs inhibitors. Shown are representative traces of the response in selected individual cells from the same experiment (gray lines, 10 cells per plot) and their average (thick black lines) (4 independent experiments were performed in total).

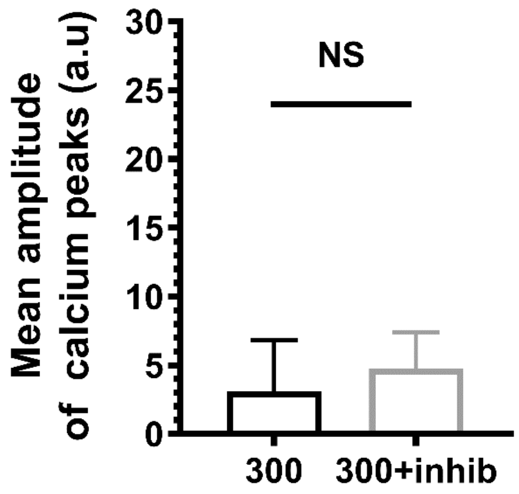

Figure S4. Effect of VOCCs inhibitors on the mean amplitude of the electro-induced Ca2+ peaks in DC3F pulsed in pure DMEM. Cells were incubated with 10 µM of Fluo-4 AM and pulsed in presence or not of VOCCs inhibitors. Data are mean ± SD (2 independent experiments with 19 cells for “300” and 24 cells for “300+inhib”). No significant effect of the inhibitors was observed, p = 0.1224 (t test). 300: one pulse of 300 V/cm (100 µs), + inhib: in the presence of 10 µM verapamil and 5 µM mibefradil.

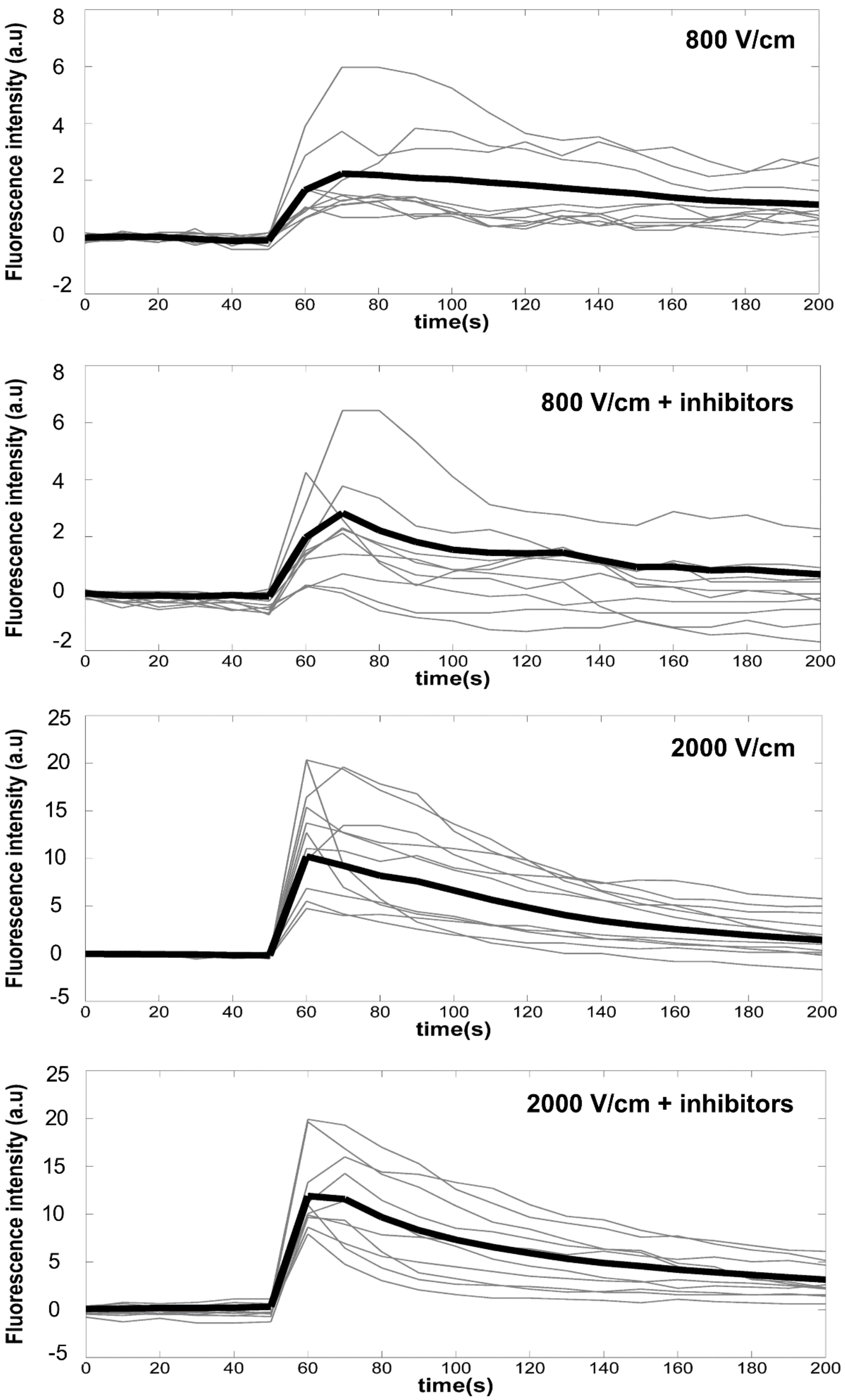

Figure S5. Typical traces of pulse-induced Ca^{2+} peaks for the haMSC in SMEM-EGTA with or without IP3R and RyR inhibitors after incubation of the cells in 10 µM fluo-4 AM. Shown are representative traces of the response in selected individual cells from the same experiment (gray lines, 10 cells per plot) and their average (thick black lines) (3 independent experiments were performed in total).

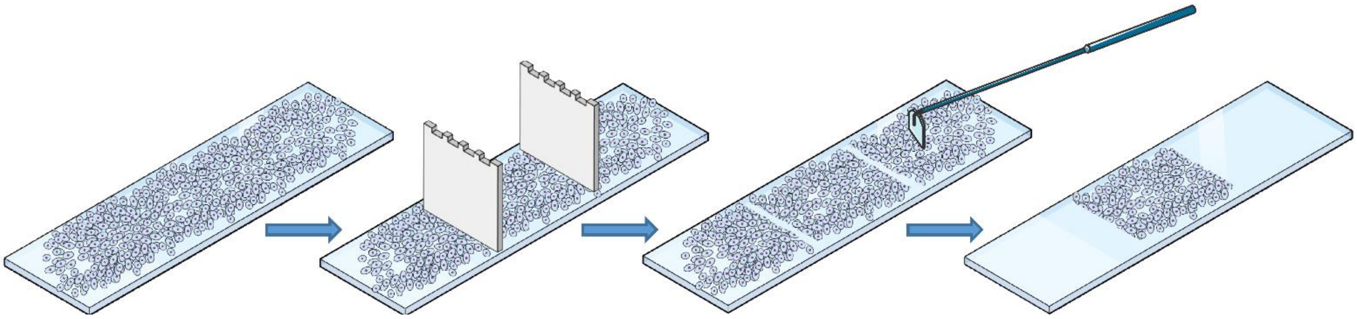

Figure S6. Protocol for cell viability assessment. Cells were seeded on 12×32 mm cover slide, one day prior to the experiments. Electric pulses were delivered using plates electrodes. The placement of the electrodes causes the loss of cells beneath the electrodes, allowing the identification of the pulsed area. Then, the non-pulsed cells were removed using a cell scraper, and the cover slide (with only the pulsed cells) analyzed for cell viability. The figure was drawn using Servier Medical Art (http://servier.com/Powerpoint-image-bank) under the Creative Commons Attribution 3.0 Unported License (https://creativecommons.org/licenses/by/3.0/).

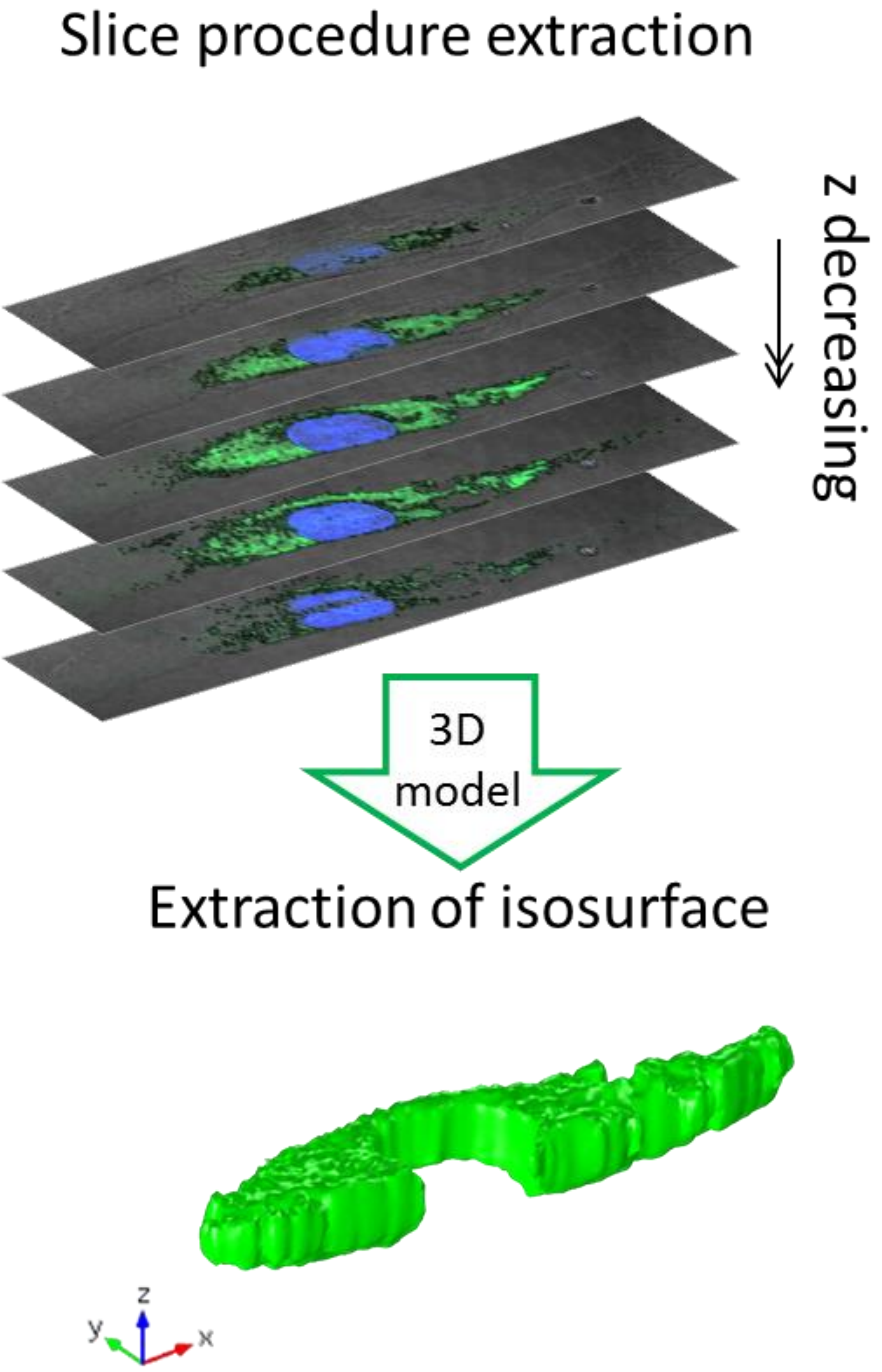

Figure S7. Illustration of ER reconstruction in MSC. For all the slices at different depths, the procedure, previously explained for green (ER) identification, was repeated. Then, each area was extruded for a thickness corresponding to the step of the microscopy procedure and finally elaborated with isosurface function in MATLABTM (v 2016) for the extraction of the 3D ER model.
